# Supplementary material for: Transcriptome Analysis of Early Senescence in the Post-Anthesis Flag Leaf of Wheat (Triticum aestivum L.)
Source: Plants (Basel). 2022 Oct 1;11(19):2593. doi: 10.3390/plants11192593 (PMC9572001; doi:10.3390/plants11192593)
Supplement: Supplementary file 1 [file plants-11-02593-s001.zip › Attached figure.pdf]

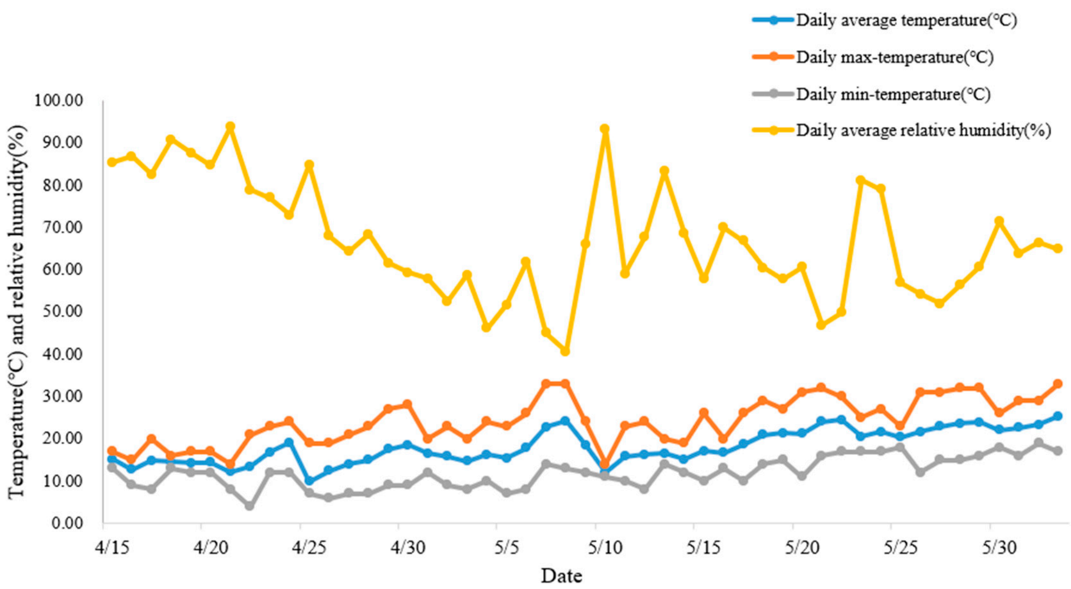

Figure S1. Daily average temperature (℃) and relative humidity (%) of wheat during flowering-maturity period. Temperature (℃) and relative humidity (%) are recorded every hour, and the value is the average of 24 values every day. (Yellow: daily average relative humidity (%). Orange: Daily max-temperature (℃). Blue: Daily average temperature (℃). Grey: Daily min-temperature(℃))

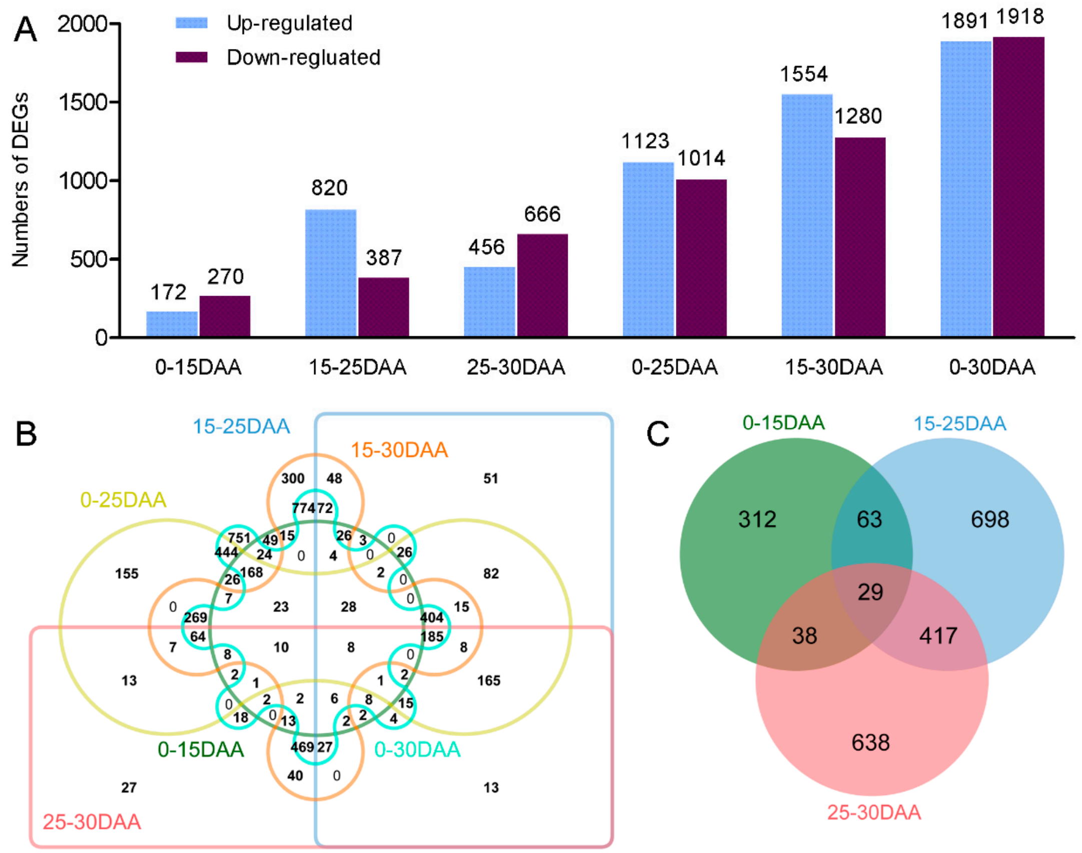

Figure S2. Number of DEGs per interval.

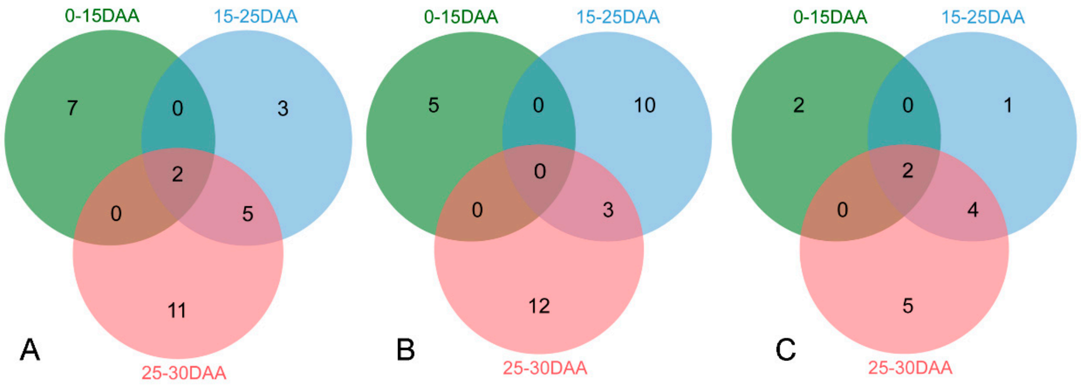

Figure S3. Metabolic pathways significantly enriched in each period. (A). All metabolic pathways significantly enriched. (B). Metabolic pathways with significant enrichment of up-regulated genes. (C). Metabolic pathways with significant enrichment of down-regulated expression genes.

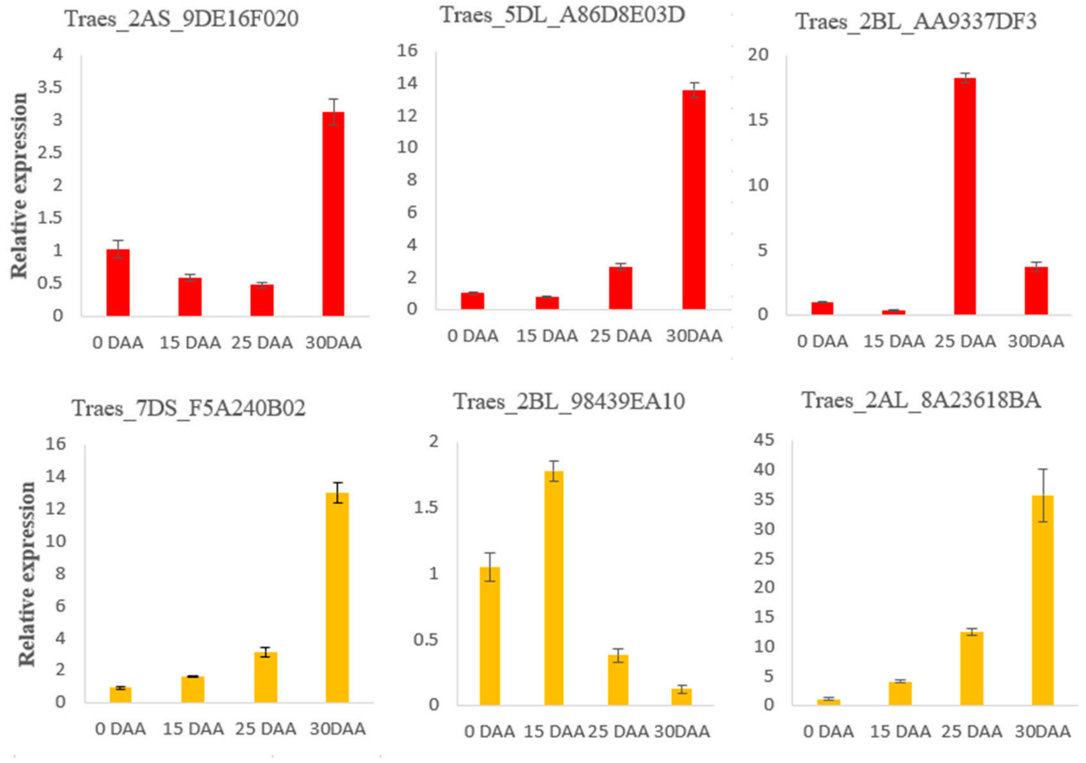

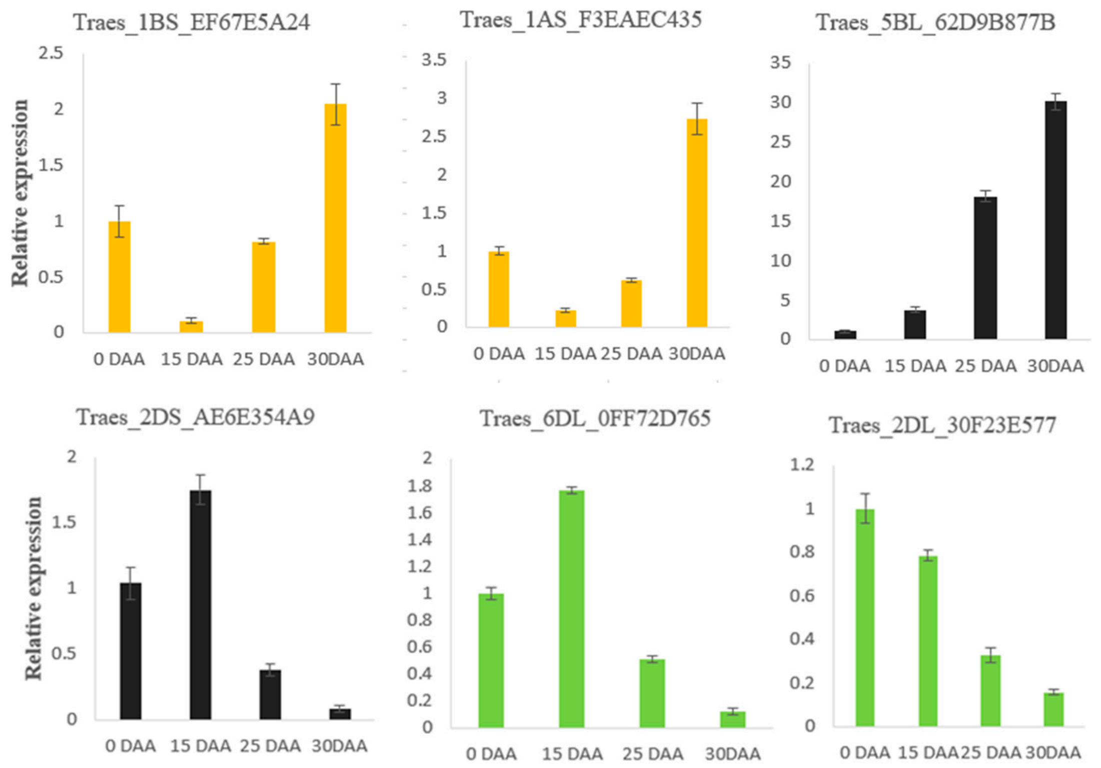

Figure S4. Analysis of relative expressions of different types of SAGs genes. Red: Transporter-related; Orange: Transcription factor-related; Black: Phytohormone-related; Green: Chlorophyll-related. The relative expression of each gene 0 DAA is '1.'

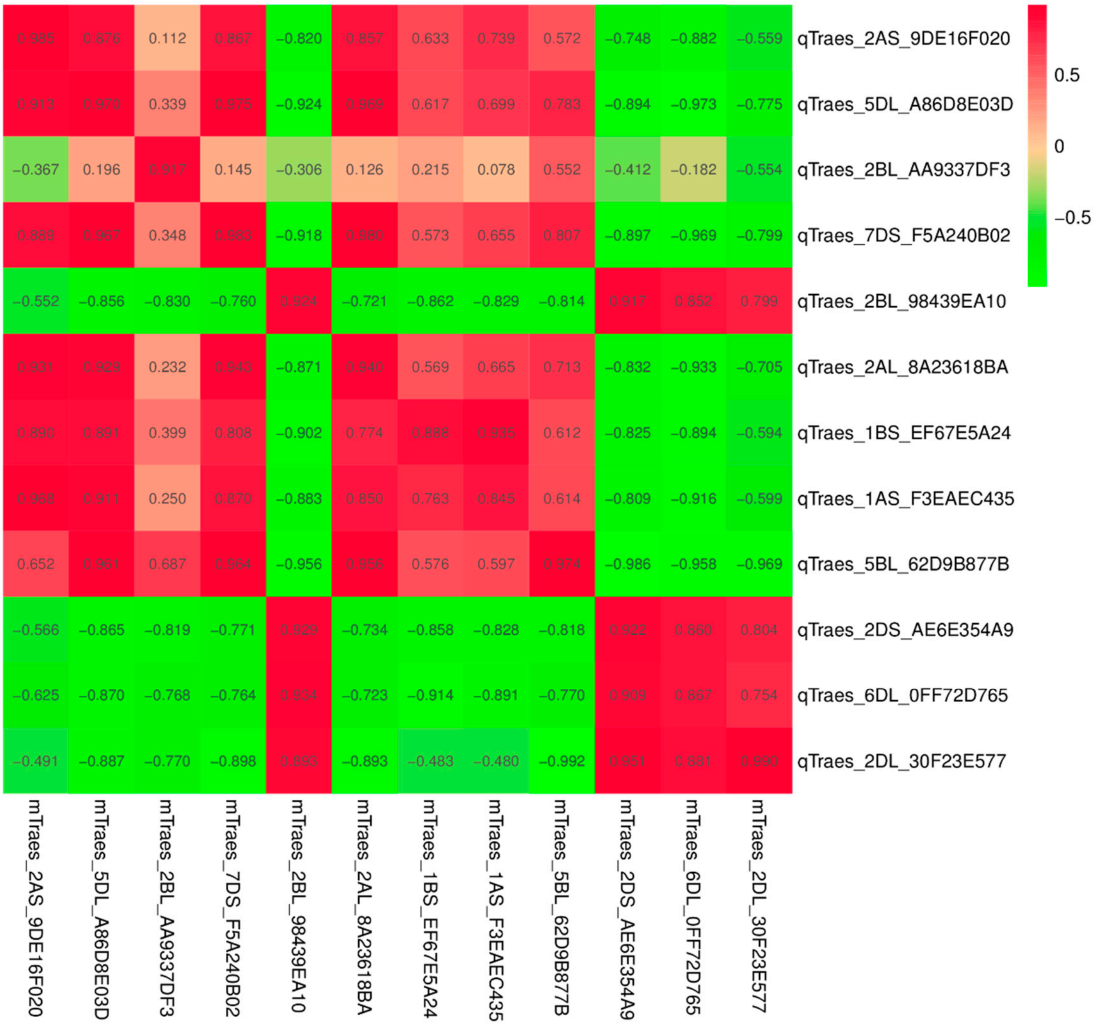

Figure S5: Heat map of RNA-seq and qRT-PCR correlation. q+gene represents qRT-PCR data; m+gene represents RNA-seq data.

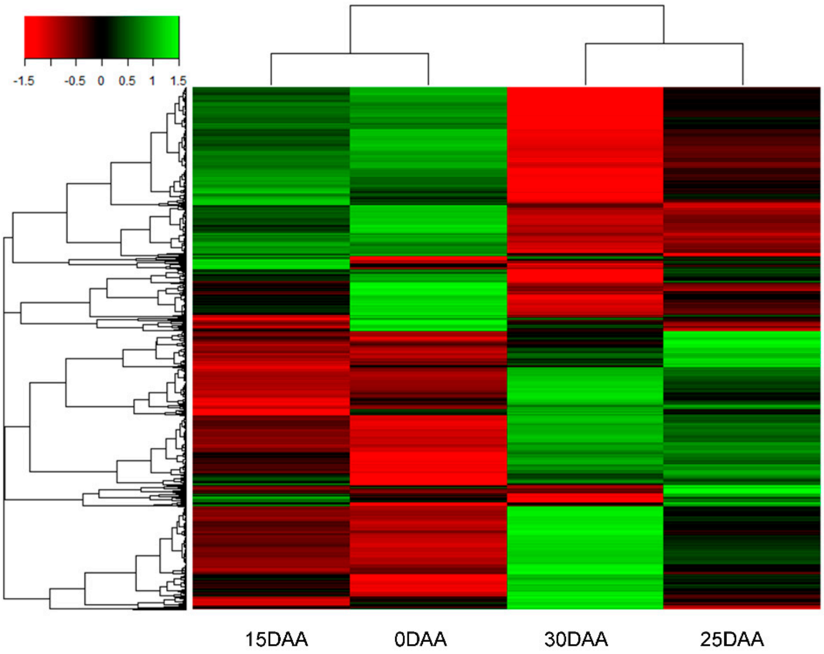

Figure S6. Clustering heat map of differentially expressed gene expression trends.

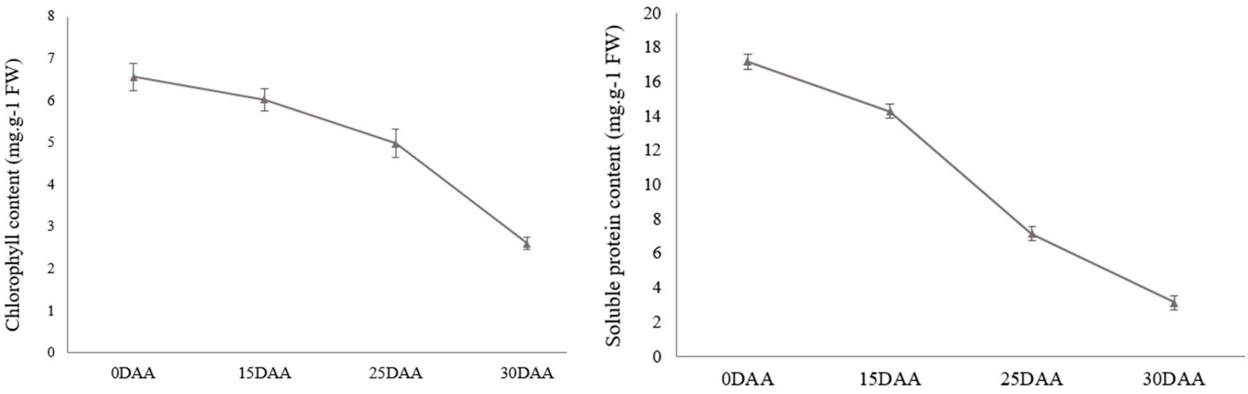

Figure S7: Changes in Chlorophyll content (a) and soluble protein content (b), after flowering in wheat flag leaves. For all values, n = 10 and the data represent the mean±sd.
